# One-year follow-up of young people with ME/CFS following infectious mononucleosis by Epstein-Barr virus

**DOI:** 10.3389/fped.2023.1266738

**Published:** 2024-01-18

**Authors:** Rafael Pricoco, Paulina Meidel, Tim Hofberger, Hannah Zietemann, Yvonne Mueller, Katharina Wiehler, Kaja Michel, Johannes Paulick, Ariane Leone, Matthias Haegele, Sandra Mayer-Huber, Katrin Gerrer, Kirstin Mittelstrass, Carmen Scheibenbogen, Herbert Renz-Polster, Lorenz Mihatsch, Uta Behrends

**Affiliations:** ^1^ MRI Chronic Fatigue Center for Young People (MCFC), Children’s Hospital, TUM School of Medicine, Technical University of Munich and Munich Municipal Hospital Schwabing, Munich, Germany; ^2^ Institute of Medical Immunology, Charité - Universitätsmedizin Berlin, Corporate Member of Freie Universität Berlin and Humboldt Universität zu Berlin and Berlin Institute of Health (BIH), Berlin, Germany; ^3^ Mannheim Institute of Public Health, Social and Preventive Medicine, University Medicine Mannheim, Heidelberg, Germany; ^4^ German Center for Infection Research (partner site Munich), Munich, Germany

**Keywords:** myalgic encephalomyelitis, chronic fatigue syndrome, infectious mononucleosis, Epstein-Barr virus, EBV, adolescents, ME/CFS, follow-up

## Abstract

**Background:**

Infectious mononucleosis after primary infection with Epstein-Barr virus (EBV-IM) has been linked to the development of myalgic encephalomyelitis/chronic fatigue-syndrome (ME/CFS) in children, adolescents, and young adults. Here, we present clinical phenotypes and follow-up data from a first German cohort of young people with ME/CFS following EBV-IM.

**Methods:**

12 adolescents and 13 young adults were diagnosed with IM-triggered ME/CFS at our specialized tertiary outpatient service by clinical criteria requiring post-exertional malaise (PEM) and a history of confirmed EBV primary infection as triggering event. Demographic information, laboratory findings, frequency and severity of symptoms, physical functioning, and health-related quality of life (HRQoL) were assessed and re-evaluated 6 and 12 months later.

**Results:**

Young adults displayed more severe symptoms as well as worsening of fatigue, physical and mental functioning, and HRQoL throughout the study, compared to adolescents. After one year, 6/12 (54%) adolescents no longer met the diagnostic criteria for ME/CFS while all young adults continued to fulfill the Canadian consensus criteria. Improvement in adolescents was evident in physical functioning, symptom frequency and severity, and HRQoL, while young adults showed little improvement. EBV serology and EBV DNA load did not correlate with distinct clinical features of ME/CFS, and clinical chemistry showed no evidence of inflammation. Remarkably, the median time from symptom onset to ME/CFS diagnosis was 13.8 (IQR: 9.1–34.9) months.

**Conclusions:**

ME/CFS following EBV-IM is a severely debilitating disease often diagnosed late and with limited responses to conventional medical care, especially in adults. Although adolescents may have a better prognosis, their condition can fluctuate and significantly impact their HRQoL. Our data emphasize that biomarkers and effective therapeutic options are also urgently needed to improve medical care and pave the way to recovery.

## 1 Introduction

Myalgic encephalomyelitis/chronic fatigue syndrome (ME/CFS) is a complex and debilitating multi-system disease characterized by fatigue, post-exertional malaise (PEM) and additional symptoms, including unrefreshing sleep, cognitive impairment, orthostatic intolerance, and/or chronic pain. Up to 25% patients are severely affected and bound to home or bed (1, 2). ME/CFS has been identified as an important cause for long-lasting school absence (3–8) and is associated with a significant reduction of health-related quality of life (HRQoL) (7, 9–11).

Pre-pandemic global prevalence estimates for ME/CFS were 0.3%–0.5%, with age peaks at onset of 11–19 and 30–39 years (12). The prevalence reported for children and adolescents ranged from 0.1% to 1.9%, depending on case definitions, geographical region, and screening methods. Up to 95% of children with ME/CFS may remain undiagnosed (13). Adolescent girls represent the majority of pediatric ME/CFS patients, with a post-pubertal female-to-male ratio of 3–4:1 (8, 13).

Infectious triggers of ME/CFS account for 23%–90% pediatric cases (4, 14–16). 80% pediatric ME/CFS patients of a large Australian cohort recalled an initial infection, and 40% an infectious mononucleosis (IM) by Epstein-Barr virus (EBV) (7). ME/CFS was reported in 13%, 7%, and 4% adolescents in the US at 6, 12, and 24 months (17, 18), and in 23% college students at 3–6 months after EBV-IM, respectively (19). While EBV was the most prominent trigger of ME/CFS until 2019 (7, 18–30), it became outranked by severe acute respiratory coronavirus type 2 (SARS-CoV2), which was estimated to cause at least a doubling of ME/CFS cases worldwide, including Germany (31–33).

The pathomechanisms of ME/CFS remain unclear. Genetic polymorphisms might contribute to pathogenic immune dysregulation (34). Emerging evidence suggests vascular changes causing hypoperfusion of muscles and brain (35). Microbiome dysbiosis, defects in energy metabolism, dysregulated hormones, and vagus nerve dysfunction have been discussed (20, 36–38). A causative role of human herpes virus (HHV) reactivation was evaluated but has not been proven yet (39–44). We recently reported, that EBV (HHV4) might initiate autoimmunity by molecular mimicry (45).

Candidate risk factors for EBV-triggered ME/CFS include disease severity and days-in-bed during the acute phase, initial pain and autonomic symptoms, lower mental health scores, higher scores for anxiety, depression, and perceived stress, female gender, as well as distinct laboratory findings (e.g., elevated C-reactive protein and cytokine levels). However, different case definitions have been used and findings were inconsistent (19, 23, 27–29). Jason and colleagues found that baseline anxiety, stress, depression, or coping skills did not predict the development of ME/CFS after EBV-IM, while preceding symptoms of the ME/CFS spectrum increased the risk (19).

ME/CFS is diagnosed according to clinical case definitions and after thorough differential diagnosis (46). In adults the Institute of Medicine (IOM) criteria (47) are recommended for screening and the Canadian Consensus Criteria (CCC) (48) for diagnosis and research. For children and adolescents the CCC were adapted in a “pediatric case definition” by Jason and colleagues (PCD-J) (49) and a “clinical diagnostic worksheet” developed by Rowe and colleagues (CDW-R) (6). All four scores require PEM. Comorbidities can include autoimmune thyroiditis, hypermobile Ehlers Danlos syndrome (hEDS), and postural orthostatic tachycardia syndrome (PoTS) (6, 46).

No specific ME/CFS treatment is available yet. Consequent self-management with pacing was recommended together with non-pharmaceutical and pharmaceutical approaches to reduce the severity and frequency of symptoms. Psychosocial support may help with implementing coping strategies, and occupational therapy can support daily life and education (6, 46, 50). Promising experimental strategies are targeting the immune, vascular, and nervous system (51).

With adequate treatment, the course of disease seems to be better in children and adolescents compared to adults, with pediatric recovery rates of 5%–83% (4, 6–8, 14–16, 26, 52–58). Recovery rates in young people have been operationalized by measuring school attendance, symptom frequency and severity, as well as fulfillment of diagnostic criteria (53, 59). In an Australian pediatric cohort, one and two thirds of the patients recovered after 5 and 10 years, respectively, with a median disease duration of 5 (1–14) years in those who recovered (7).

However, in many cases the course of ME/CFS is fluctuating, with periods of deterioration (“crashes”), stabilization, improvement, or relapse-remitting cycles (6, 7, 22). About 40% of adult patients are estimated to improve over time, but only 5% fully recover (60, 61). Inferior outcomes might in part be due to inappropriate management resulting from inadequate disease-specific knowledge of medical staff (3, 52, 62, 63), to a lack of medical services and barriers to the health care system for patients with ME/CFS (64–66), and to stigmatization.

Here, we present a first German cohort of young ME/CFS patients diagnosed after confirmed EBV-IM at our MRI Chronic Fatigue Center for Young People (MCFC) and participating in our prospective MUC-CFS studies. The MCFC, so far, is Germany's sole pediatric university center specialized on ME/CFS research and care. The MUC-CFS studies offer comprehensive insights into patient demographics, clinical phenotypes, and health-related quality of life (HRQoL) at diagnosis and during follow-up. Our primary objective was to assess disease trajectories at 6 and 12 months after ME/CFS diagnosis to explore potential age-sepcific differences.

## 2 Methods

### 2.1 Study population, diagnostic work-up, and standard treatment

A cohort of 12 adolescents and 13 adults was diagnosed with ME/CFS after confirmed EBV-IM from March 2019 to November 2022 at our tertiary pediatric university hospital, enrolled in our single-center prospective MUC-CFS cohort studies, and reassessed at 6 and 12 months. Confirmed EBV-IM was defined as a combination of typical symptoms (e.g., fever, fatigue, sore throat, lymphadenopathy, and/or splenomegaly) and typical serology (positive IgM and/or IgG antibodies against EBV viral capsid antigen (VCA) without IgG antibodies against EBV nuclear antigen 1 (EBNA-1), in some cases with documented subsequent EBNA-1-IgG seroconversion). Diagnostic ME/CFS criteria were applied depending on age: For adults (≥18 years) the CCC were used. Adolescents needed to meet either the CCC or the less strict CDW-R criteria, with a disease duration of at least 3 or 6 months, respectively. PEM had to last for more than 14 h after mild exertion. All patients underwent a thorough differential diagnostic work-up (laboratory analyses, ECG, UCG, EEG, cMRI, pulmonary function analyses, psychological evaluation, additional investigations depending on symptoms) as recommended (6). A 10-minute passive standing test screened for orthostatic intolerance (OI), PoTS, or orthostatic hypotonia (OH). All patients received a symptom-oriented, non-pharmaceutical and/or pharmaceutical treatment, were guided on self-management, and were provided with psychosocial support, including adapted school education and home care if needed.

### 2.2 Data collection

Clinical data were collected from clinical records and questionnaires. For personal or telephone follow-up visits, questionnaires were mailed to the families one month in advance. Five well-established patient-reported outcome measures (PROM) were used: (i) The Pediatric Quality of Life Inventory (PedsQL) was used to assess HRQoL in pediatric patients. It comprises 20 items and four subscales, namely physical, emotional, social, and school functioning, with good internal consistency and reliability (67). (ii) The Short Form-36 Health Survey (SF-36) is a well-validated 36-item questionnaire for measuring HRQoL in people older than 13 years, with eight subscales (physical functioning, role physical, general health, bodily pain, social functioning, vitality, role emotional, and mental health) ranging from 0 to 100. Lower scores indicate more impairment (68). (iii) The Chalder Fatigue Scale (CFQ) measures physical and mental fatigue and consists of eleven items on a Likert scale from 0 to 3. The total score ranges from 0 to 33, with 33 indicating most severe fatigue (69). (iv) The Charité Symptom Inventory (CSI), adapted from the CDC Symptom Inventory, rates frequency and severity of typical symptoms of ME/CFS within the month prior to the visit. Scales rate from 0 (not present) to 3 (severe) for severity and from 0 (not present) to 4 (always) for frequency of symptoms (70). (v) The Bell Score assesses the severity of ME/CFS by evaluating the impairment of daily activities (71); for adolescents the wording was adapted (e.g., “school” instead of “work”).

### 2.3 Statistical analyses

Statistical analyses utilized R version 4.2.1 (“Funny-Looking Kid”) (72). Categorial variables were compared using Fisher's exact test or Pearson's *χ*^2^ test. Numeric variables were compared between groups using the Wilcoxon rank-sum or Kruskal–Wallis test, as appropriate. Spearman's rank coefficient assessed correlations. Cox regression analysed association between independent variables and the time-to first presentation in the MCFC. Repeated measures correlation gauged within-subject PROMs' correlation (73). Due to small sample size and no adjustment for multiple testing, all *P*-values were considered exploratory. Significance level was set to *α* = 0.05.

## 3 Results

### 3.1 Baseline demographics and clinical characteristics

Baseline characteristics are shown in Table 1. All 25 patients (80% female) had a history of EBV-IM with typival symptoms and documented serological findings indicating EBV primary infection at the time of disease onset. Adolescents (48%, median age at onset 15, IQR 13–15) did not differ from young adults (≥18 years) (52%, median age at onset 10, IQR: 18–21) with regard to demographics, medical and family history, and current medical care. The median time between EBV-IM and ME/CFS diagnosis at the first visit was 13.8 months (range 4–84), with no significant difference between males and females (*P* = 0.272), and/or adults and adolescents (*P* = 0.596). The time delay from symptom onset to diagnosis was less than 6, 12, and 24 months in 1/13 (8%), 5/13 (38%), and 7/13 (54%) adults as well as in 1/12 (8%), 5/12 (42%), and 10/12 (83%) adolescents (Supplementary Figure S1).

**Table 1:** Baseline demographics and clinical characteristics of the cohort.

Characteristics	All	Adults	Adolescents	*P*-value^a^
Number of patients	*n* = 25	*n* = 13	*n* = 12	
Age and illness duration^b^				
Age at first visit	18 (16–21)	21 (19–22)	16 (14–16)	<0.001
Age at onset	16 (14–19)	19 (18–21)	15 (13–15)	<0.001
Illness duration in months from onset to first visit	13.8 (9.1–34.9)	16.9 (9.4–44.1)	13.2 (8.8–22.0)	0.503
Baseline questionnaire results				
Chalder fatigue scale^c^	25 (5)	28 (4)	22 (5)	**0** **.** **006**
Bell Score^b^	40 (30–50)	30 (30–40)	50 (40 –50)	0.056
SF-36 PCS^c^	29 (9)	26 (8)	32 (9)	0.151
SF-36 MCS^c^	42 (11)	39 (12)	45 (9)	0.211
PedsQL^c^	46 (14)	35 (11)	54 (9)	**0**.**002**
Gender^d^				
Female	20/25 (80)	11/13 (85)	9/12 (75)	0.645
ME/CFS criteria^d^				
CCC	21/25 (84)	13/13 (100)	8/12 (67)	**0**.**039**
CDW-R	12/12 (100)	N/A	12/12 (100)	0.077
Comorbidity^d^				
PoTS	19/23 (83)	11/11 (100)	8/10 (80)	>0.999
Allergies	12/25 (48)	7/13 (54)	5/12 (42)	0.543
Asthma	1/25 (4)	0/13 (0)	1/12 (8)	>0.999
Neurodermatitis	1/25 (4)	1/13 (8)	0/12 (0)	>0.999
Psychiatric disorder	3/25 (12)	2/13 (15)	1/12 (8)	>0.999
Medical history^d^				
Trauma/surgery	1/25 (4)	0/13 (0)	1/12 (8)	0.480
Asthma	2/25 (8)	1/13 (8)	1/12 (8)	>0.999
Psychiatric disorder	1/25 (4)	0/13 (0)	1/12 (8)	0.480
Current medical care^d^				
Complete vaccinations	22/22 (100)	11/11 (100)	11/11 (100)	
Nutrition supplements	17/24 (71)	10/12 (83)	7/12 (58)	0.319
Prescription medication	11/24 (46)	5/12 (42)	6/12 (50)	>0.999
Degree of disability	1/25 (4)	0/13 (0)	1/12 (8)	>0.999
Medical aid	0/25 (0)	0/13 (0)	0/12 (0)	
Long-term care level	0/25 (0)	0/13 (0)	0/12 (0)	
Family history^d^				
ME/CFS in family	2/25 (8)	1/13 (8)	1/12 (8)	>0.999
AID in family	10/25 (40)	6/13 (46)	4/12 (33)	0.688
PID in family	0/25 (0)	0/13 (0)	0/12 (0)	
IM in family	16/25 (64)	9/13 (69)	7/12 (58)	0.688

CCC, Canadian consensus criteria (47); CDW-R, clinical diagnostic worksheet (6); PoTS, postural orthostatic tachycardia syndrome; AID, autoimmune disease; PID, primary immune deficiency; IM, infectious mononucleosis; PedsQL, pediatric quality of life inventory; SF-36 PCS, short form 36 health survey physical component summary score; SF-36 MCS, short form 36 health survey mental health component summary score; N/A, not applicable.

Bold values denote statistical significance at the *P* < 0.05 level.

^a^ Fisher's exact test; Wilcoxon rank sum test; Pearson's χ^2^ test.

^b^ Median (IQR).

^c^ Mean (SD).

^d^ Number of patients with indicated characteristic/number of patients investigated (%).

All adults met the CCC and all adolescents the CDW-R criteria and/or CCC, as required. Adults did not significantly differ from adolescents with regard to the baseline Bell Score or the SF-36 physical (PCS) and mental health component summary score (MCS). However, adults showed significantly higher CFQ scores (adults: 28 ± 4; adolescents: 22 ± 5; *P* = 0.006), and significantly lower PedsQL values (adults: 35 ± 11; adolescents: 54 ± 9; *P* = 0.002) compared to adolescents. At the time of diagnosis, all adolescents reported school absences, 2/11 (18%) received complementary home schooling and none had distance schooling. One patient reported a documented degree of disability, and none had received medical care at home.

24/25 (96%) patients showed comorbidities, with PoTS in 21/23 (83%) and allergies in 12/23 (48%) patients. Two patients droped out of the 10-min passive standing test due to severe OI symptoms. One patient presented with a diagnosis of anxiety disorder, and two with a mixed anxiety and depressive disorder. 17/24 (71%) patients took various nutritional supplements, and 11/24 (46%) prescription-only medications, including three patients on antidepressants. With regard to the family's medical history, in 2/25 (8%) cases ME/CFS was reported. 16/25 (64%), 10/25 (40%), and 18/25 (72%) patients remembered a family member with EBV-IM, autoimmune diseases, or either one.

The cohort had consulted several (median 6, range 1–11) private practice doctors across five different specialties (range 1–11) for ME/CFS symptoms. 11/20 (55%) patients had visited at least one hospital. 7/20 (35%) had consulted a psychotherapist/psychologist, 9/20 (45%) a naturopath, 6/20 (30%) traditional Chinese medicine, 4/20 (20%) homeopathy, and 5/20 (25%) osteopathy.

### 3.2 Baseline laboratory findings

Laboratory findings at the time of diagnosis were primarily unremarkable, without significant differences between adolescents and adults (Table 2). Besides low vitamin D levels in 14/24 (58%) patients (range 7–29 ng/ml), the most frequent laboratory findings were elevated antinuclear antibodies (ANA) present in 14/25 (56%) (range 1:100–1:800), elevated IgE in 7/25 (28%) and mild anemia in 4/25 (16%) cases. ANA titers were in the range of <1:160 in 2/6 (33%) adolescents, of 1:160–1:640 in 2/6 (33%) adolescents and 8/8 (100%) adults, and of ≥1:640 in 2/6 (33%) adolescents, with higher ANA titers compared to adults (*P* = 0.015). ANA titers did not significantly correlate with disease severity (Bell Score: *P* = 0.452; SF-36 PF: *P* = 0.858), were not significantly different between males and females (*P* = 0.521), and not associated with any sign of connective tissue disorders. Herpes simplex virus coinfection was not more frequent in adults compared to adolescents (*P* > 0.999). Neither total immunoglobulin serum levels nor phenotypes of peripheral blood lymphocytes revealed any evidence of primary immunodeficiency (PID) (Supplementary Table S1).

**Table 2:** Selected laboratory results at baseline visit.

Laboratory parameter	All *n*/*n* (%)^a^	Adults *n*/*n* (%)^a^	Adolescents *n*/*n* (%)^a^	*P*-value^b^
Blood count				
Neutropenia (<1,500/ul)	2/25 (8)	1/13 (8)	1/12 (8)	>0.999
Lymphocytes ↑	5/25 (20)	2/13 (15)	3/12 (25)	0.645
Thrombocytes ↓	0/25 (0)	0/13 (0)	0/12 (0)	
Hemoglobin ↓	4/25 (16)	1/13 (8)	3/12 (25)	0.322
Inflammation				
Sedimentation rate ↑	1/21 (5)	0/13 (0)	1/8 (12.5)	0.350
C-reactive protein ↑	0/25 (0)	0/13 (0)	0/12 (0)	
Ferritin ↑	2/23 (9)	0/13 (0)	2/10 (20)	0.178
Liver function				
GOT ↑	1/25 (4)	0/13 (0)	1/12 (8)	0.480
GPT ↑	2/25 (8)	0/13 (0)	2/12 (17)	0.220
Bilirubin ↑	1/24 (4)	1/13 (8)	0/11 (0)	>0.999
Immunoglobulins (Ig)				
IgA ↓↑	0/25 (0)	0/13 (0)	0/12 (0)	
IgM ↓↑	0/25 (0)	0/13 (0)	0/12 (0)	
IgG ↓↑	0/25 (0)	0/13 (0)	0/12 (0)	
IgE ↑	7/25 (28)	3/13 (23)	4/12 (33)	0.673
Infection Serology				
Cytomegalovirus IgG	3/25 (12)	2/13 (15)	1/12 (8)	>0.999
Herpes simplex virus IgG	3/25 (12)	2/13 (15)	1/12 (8)	>0.999
Toxoplasma IgG	1/25 (4)	1/13 (78)	0/12 (0)	>0.999
Borrelia IgG	1/25 (4)	0/13 (0)	1/12 (8)	0.480
Autoantibodies				
ANA ↑	14/25 (56)	8/13 (62)	6/12 (50)	0.561
ANCA ↑	1/25 (4)	1/13 (8)	0/12 (0)	>0.999
Anti-dsDNA ↑	0/25 (0)	0/13 (0)	0/12 (0)	
Endocrinology				
Cortisol ↓	1/25 (4)	1/13 (18)	0/12 (0)	0.480
ACTH ↑	1/23 (4)	0/12 (0)	1/11 (9)	0.478
25-OH-Vitamin-D ↓	14/24 (58)	6/12 (50)	8/12 (67)	0.680

↑above normal range; ↓ below normal range; ANA, antinuclear antibodies; ANCA, anti-cytoplasmatic antibodies; anti-dsDNA, anti-double strand DNA.

^a^ Number of patients with indicated laboratory parameter/number of patients investigated (%).

^b^ Fisher's exact test; Pearson's χ^2^ test.

Results from EBV serology and real-time PCR at the first visit are displayed in Table 3 and did not differ significantly between adolescents. No EBV DNA was detected in plasma. 8/20 (40%) patients showed EBV DNA in peripheral blood cells (5/8 very low titers, 1/8 17.7 Geq/10^5^, 1/8 70.1 Geq/10^5^, and 1/8 121.8 Geq/10^5^), and 14/25 (66%) in throat washes. EBV DNA load in throat washes did not significantly correlate with disease severity (Bell Score: *P* = 0.686; SF-36 PCS: *P* = 0.871). All patients showed anti-EBV-VCA IgG as expected, 23/25 (92%) had detectable anti-EBNA-1 IgG and 6/25 (24%) anti-EBV-VCA IgM. The detection of anti-EBV-VCA IgM did not significantly correlate with disease severity (Bell Score: *P* = 0.877; SF-36 PCS: *P* = 0.788). Results of EBV immunoblots revealed IgG antibodies against early antigens (EA) p54 and p138, the immediate early antigen BZLF1, virus capsid antigens (VCA) p23 and p18, and EBNA-1 in 8/25 (32%), 5/25 (20%), 18/25 (72%), 23/25 (92%), 24/25 (96%), and 22/25 (88%) patients, respectively.

**Table 3:** EBV serology, PCR and IgG immunoblot at baseline visit.

EBV diagnostics	All *n*/*n* (%)^a^	Adults *n*/*n* (%)^a^	Adolescents *n*/*n* (%)^a^	*P*-value^b^
EBV PCR				
DNA in cell fraction				0.927
–	12/20 (60)	7/12 (58)	5/8 (62)	
(+)	5/20 (25)	3/12 (25)	2/8 (25)	
+	3/20 (15)	2/12 (17)	1/8 (12)	
DNA in plasma				
–	25/25 (100)	13/13 (100)	12/12 (100)	
+	0/25 (0)	0/12 (0)	0/12 (0)	
DNA in throat wash				>0.999
–	11/25 (44)	6/13 (46)	5/12 (42)	
+	14/25 (66)	7/13 (54)	7/12 (54)	
EBV ELISA				
VCA IgM				0.110
–	18/25 (72)	7/13 (54)	11/12 (92)	
(+)	1/25 (4)	1/13 (8)	0/12 (0)	
+	6/25 (24)	5/13 (38)	1/12 (8)	
VCA IgG				
–	0/25 (0)	0/13 (0)	0/12 (0)	
+	25/25 (100)	13/13 (100)	12/12 (100)	
EBNA1 IgG				0.220
–	2/25 (8)	0/13 (0)	2/12 (17)	
+	23/25 (92)	13/13 (100)	10/12 (83)	
EBV IgG Immunoblot				
EAp54				0.282
–	14/25 (56)	7/13 (54)	7/12 (58)	
(+)	3/25 (12)	3/13 (23)	0/12 (0)	
+	8/25 (32)	3/13 (23)	5/12 (42)	
EAp138				0.233
–	14/25 (56)	6/13 (46)	8/12 (67)	
(+)	6/25 (24)	5/13 (38)	1/12 (8)	
+	5/25 (20)	2/13 (15)	3/12 (25)	
BZLF1				0.293
–	3/25 (12)	3/13 (23)	0/12 (0)	
(+)	4/25 (16)	2/13 (15)	2/12 (17)	
+	18/25 (72)	8/13 (62)	10/12 (83)	
VCAp23				>0.999
–	2/25 (8)	1/13 (8)	1/12 (8)	
(+)	0/25 (0)	0/13 (0)	0/12 (0)	
+	23/25 (92)	12/13 (92)	11/12 (92)	
VCAp18				0.480
–	1/25 (4)	0/13 (0)	1/12 (8)	
(+)	0/25 (0)	0/13 (0)	0/12 (0)	
+	24/25 (96)	13/13 (100)	11/12 (92)	
EBNA-1				0.344
–	2/25 (8)	0/13 (0)	2/12 (17)	
(+)	1/25 (4)	1/13 (8)	0/12 (0)	
+	22/25 (88)	12/13 (92)	10/12 (83)	

EBV, Epstein-Barr virus; VCA, virus capsid antigen; EBNA, EBV nuclear antigen; EA, early antigen.

^a^ Number of patients with indicated laboratory parameter/number of patients investigated (%).

^b^ Fisher's exact test.

### 3.3 ME/CFS criteria

**Figure 1:**
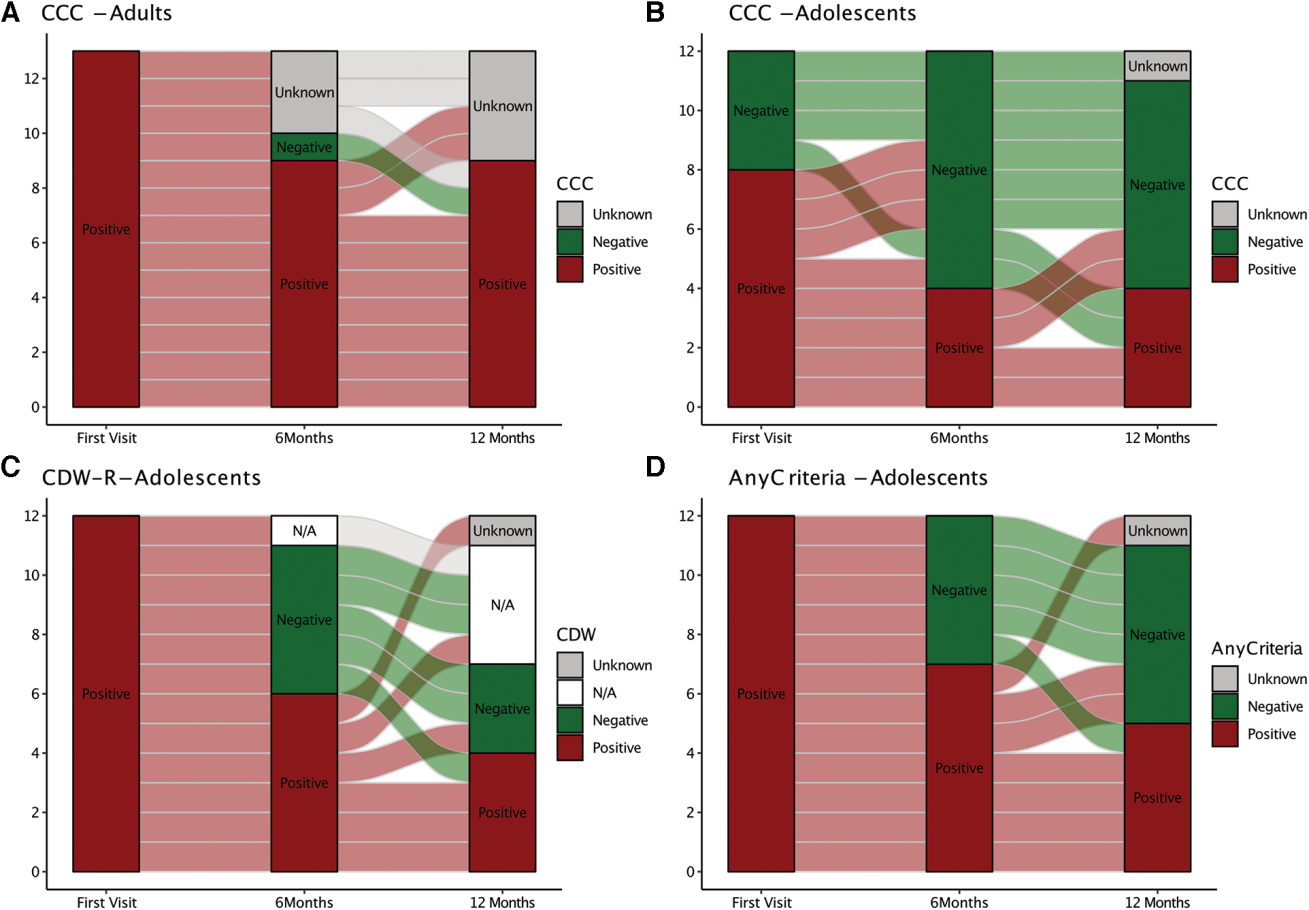
Alluvial chart illustrating ME/CFS diagnostic criteria fulfillment over time. The chart depicts diagnostic criteria fulfillment (red) or non-fullfillment (green) at the first visit and at 6 and 12 months. (Non-)fullfillment of the Canadian Consensus Criteria (CCC) is shown for adults (**A**). (Non)-fullfillment of CCC only (**B**), Rowe's diagnostic worksheet (CDW-R) criteria only, (**C**) or either of both (CCC or CDW-R) (**D**) is shown for adolescents. CDW-R criteria were not applicable anymore (N/A) when adolescents had turned 18 years.

Follow-up data were available at 6 months after ME/CFS diagnosis from 22/25 (88%) patients, including 10/13 (77%) adults and 12/12 (100%) adolescents, and at 12 months from 20/25 (80%) patients, including 9/13 (69%) adults and 11/12 (92%) adolescents. Reasons for drop out were recovery (one adolescent), worsening of symptoms (one adult), or unknown (two patients). Changes in CCC and CDW-R criteria fulfilment are shown in Figure 1. Seven adults fulfilled the CCC at all three visits. One became CCC negative at 6 months but met the CCC criteria again at 12 months (Figure 1A). Six adolescents were still positive for the CDW-R criteria at 6 months and only four at 12 months follow-up. One patient became CDW-R negative at 6 months but met the CDW-R criteria again at 12 months (Figure 1B). By 6 months one and by 12 months three additional pediatric patients had turned 18 years old, and therefore the CDW-R criteria were not applicable anymore (indicated by N/A in Figure 1C). The CCC criteria were fulfilled by 8/12 (67%), 4/12 (33%), and 4/11 (36%) adolescents at the first visit, 6 months and 12 months. Two adolescents who were CCC positive at 12 months had been negative at the previous visits (Figure 1C). 7/12 (58%) adolescents met either the CCC or the CDW-R criteria at 6 months, and 5/11 (45%) either of both at 12 months (Figure 1D). Patients with partial recovery still presented with some of the symptoms. Two patients reported on OI only, one on fatigue with limitations in daily life and headaches, and three on several symptoms without fatigue. All patients in partial remission were adolescents (*P* = 0.005) and had a relatively short illness duration of less than three years (mean 24 months, range 15–34 months). They had significantly less fatigue (CFQ Likert score: *P* = 0.001) and higher HRQoL (PedsQL: *P* = 0.026) at diagnosis compared to patients without partial remission (Supplementary Table S2). Patients in partial remission did not significantly differ in any of the other baseline characteristics and laboratory parameters tested, including EBV antibodies and DNA (Supplementary Tables S2,S3).

### 3.4 Number, frequency, and severity of symptoms

**Figure 2:**
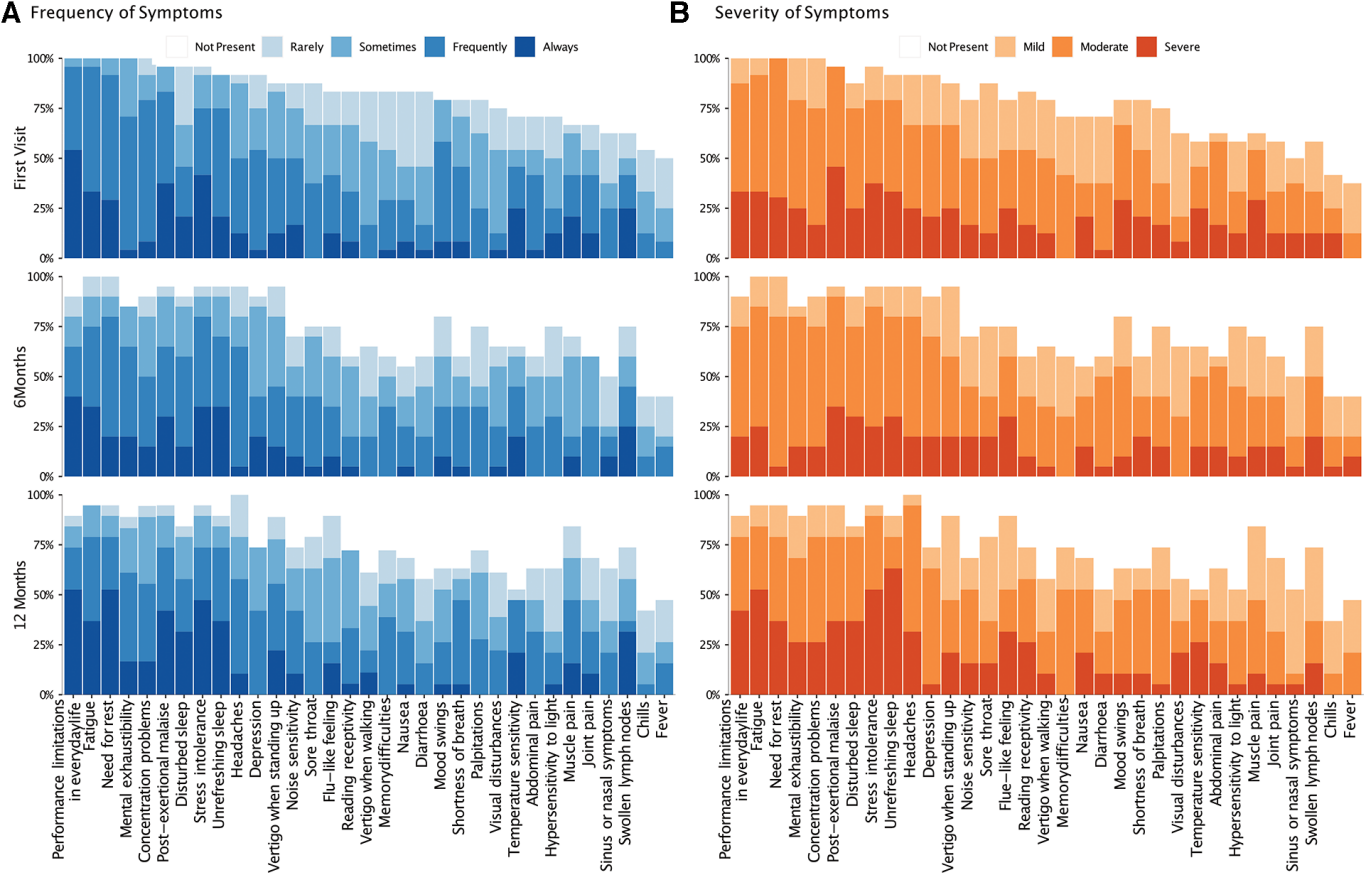
Frequency and severity of symptoms over time. The bar-chart displays individual symptoms on the *x*-axis. The *y*-axis shows the frequency (**A**) and severity (**B**) of symptoms on the left and right, respectively. The severity scale for each symptom ranged from 0 (not present) to 4 (severe), and the frequency scale from 0 (not present) to 5 (always present). At each time point, the chart shows the proportion of patients reporting the relevant symptom, with rating of severity and frequency rating, indicated by color-code.

At the baseline visit, patients presented with 27 ± 5 symptoms (mean ± SD), with 15 ± 5 occurring at least frequently (Figure 2). The symptoms reported at least frequently (3 or 4 on Likert scale) included fatigue (96%), limitations in daily life (96%), need for rest (92%) and PEM (83%). The most common severe (3 on Likert scale) symptoms were PEM (46%), stress intolerance (38%), fatigue (33%), limitations in daily life (33%), and unrefreshing sleep (33%). The number, severity, and frequency of individual symptoms did not significantly change between the first and follow-up visits (Supplementary Table S4). Adults reported slightly more symptoms (29 ± 3) than adolescents (25 ± 7, *P* = 0.084). Symptoms occurring at least frequently were more common in adults than adolescents (19 ± 6 vs. 12 ± 3, *P* = 0.006). This difference was also evident at the follow-up visits (Supplementary Table S5).

### 3.5 Patient-reported outcome measures

**Figure 3:**
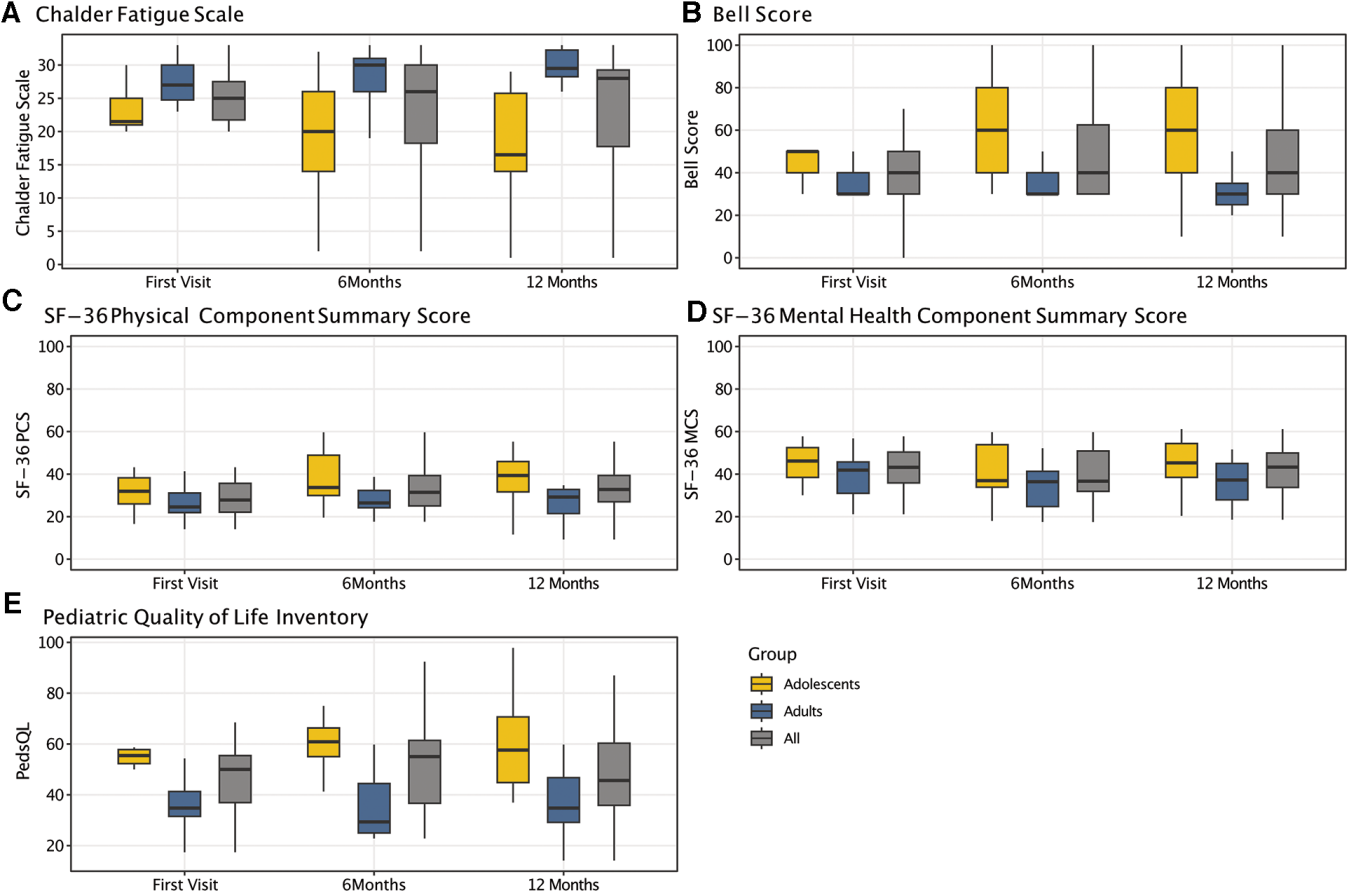
Results of patient-reported outcome measures over time. Boxplots displaying the dynamics of results from the chalder fatigue scale (CFQ) (**A**), the bell score (**B**), the SF-36 physical (**C**) (PCS) and mental health component summary score (**D**) (MCS), and the pediatric quality of life inventory (**E**) (PedsQL) for the entire cohort as well as for adolescents and adults only, respectively.

At the first visit, the CFQ Likert score of the cohort was 25 ± 5 and did not significantly change over time. While adults showed a moderate worsening from the first (28 ± 4) to follow-up visits (28 ± 4 at 6months, 29 ± 4 at 12-months), adolescents demonstrated a moderate improvement (22 ± 5 at first visit, 19 ± 9 at 6 months, 18 ± 9 at 12-months) (Table 4 and Figure 3A). At all visits, adolescents had significantly less fatigue than adults (first visit: *P* = 0.006; 6-months: *P* = 0.016; 12 months: *P* = 0.003) (Supplementary Table S6).

**Table 4:** Patient-reported outcome measures of the cohort.

	All (*n* = 25)				Adults (*n* = 13)				Adolescents (*n* = 12)			
	First Visit	6 Months	12 Months	*P*-value^a^	First Visit	6 Months	12 Months	*P*-value^a^	First Visit	6 Months	12 Months	*P*-value^a^
Bell score^b^	40 (30, 50)	40 (30, 63)	40 (30, 60)	0.384	30 (30, 40)	30 (30, 40)	30 (25, 35)	0.368	50 (40, 50)	60 (40, 80)	60 (40, 80)	0.232
PedsQL^c^												
Total score	46 (14)	50 (19)	50 (21)	0.718	35 (11)	37 (15)	37 (15)	0.968	54 (9)	62 (14)	61 (20)	0.368
Physical	40 (18)	46 (24)	40 (26)	0.748	30 (12)	29 (13)	25 (15)	0.842	48 (18)	60 (22)	53 (27)	0.531
Psychosocial	49 (14)	53 (18)	56 (20)	0.698	38 (13)	41 (17)	45 (19)	0.695	58 (6)	63 (14)	64 (18)	0.719
School	33 (14)	43 (22)	47 (28)	0.115	29 (18)	37 (22)	33 (26)	0.871	35 (11)	48 (23)	58 (25)	**0** **.** **030**
Social	66 (21)	64 (20)	64 (20)	0.919	50 (18)	49 (15)	51 (15)	0.916	77 (16)	76 (16)	76 (16)	0.944
Emotional	50 (21)	49 (24)	53 (25)	0.843	34 (15)	36 (23)	44 (21)	0.357	61 (17)	60 (19)	60 (27)	0.921
SF-36^c^												
Physical functioning	54 (25)	60 (26)	55 (34)	0.792	42 (21)	46 (20)	35 (27)	0.806	65 (25)	71 (25)	73 (31)	0.581
Role physical	12 (15)	18 (33)	22 (37)	0.897	7 (12)	3 (8)	6 (11)	0.677	17 (16)	30 (40)	38 (46)	0.843
Bodily pain	41 (26)	49 (27)	49 (29)	0.433	34 (21)	34 (15)	37 (23)	0.929	47 (29)	62 (29)	60 (31)	0.335
General health	26 (12)	28 (15)	29 (17)	0.865	22 (11)	21 (9)	20 (7)	0.969	30 (12)	35 (15)	37 (20)	0.685
Vitality	22 (14)	29 (22)	27 (26)	0.737	15 (13)	18 (11)	11 (8)	0.481	29 (12)	38 (24)	42 (28)	0.447
Social functioning	41 (27)	43 (33)	41 (35)	0.980	31 (23)	24 (22)	24 (25)	0.520	51 (27)	59 (32)	58 (36)	0.636
Role emotional	71 (39)	55 (41)	65 (44)	0.444	64 (46)	52 (44)	56 (47)	0.824	78 (33)	58 (40)	73 (41)	0.383
Mental health	59 (19)	54 (22)	59 (21)	0.756	52 (20)	43 (20)	51 (18)	0.471	65 (17)	63 (19)	66 (21)	0.923
Physical component summary score	29 (9)	34 (12)	32 (13)	0.434	26 (8)	28 (8)	25 (9)	0.839	32 (9)	38 (13)	38 (13)	0.302
Mental health component summary score	42 (11)	39 (13)	41 (12)	0.589	39 (12)	34 (11)	37 (11)	0.631	45 (9)	42 (14)	45 (12)	0.817

PedsQL, pediatric quality of life inventory; SF-36, short form 36 health survey.

Bold values denote statistical significance at the *P* < 0.05 level.

^a^ Kruskal–Wallis rank sum test.

^b^ Median (IQR).

^c^ Mean (SD).

The median Bell Score was 40 (IQR: 30–50) and did not significantly change over time (*P* = 0.384), with a median adults' Bell Score of 30 at all visits. The adolescents' Bell Score moderately but not significantly improved from the first (median: 50, IQR: 40–50) to follow-up visits (both median: 60, IQR: 40–80) (*P* = 0.232) (Table 4 and Figure 3B). It was significantly better than adults' Bell Score at all visits (first visit: *P* = 0.019; 6 months: *P* = 0.019; 12 months: *P* = 0.007) (Supplementary Table S6).

The SF-36 summary and subscales did not significantly change between visits. However, adolescents had a significantly better PCS at the 12 months than adults (*P* = 0.013) (Table 4 and Figures 3C,D). Compared to adults, adolescents were significantly better at the first visit with regard to physical functioning (*P* = 0.039) and vitality (*P* = 0.012), at 6 months to physical functioning (*P* = 0.039), pain (*P* = 0.039), general health (*P* = 0.032), social (*P* = 0.025), and mental health (*P* = 0.025), and at 12 months to physical functioning (*P* = 0.019) and vitality (*P* = 0.010). There was no significant difference between adults and adolescents with regard to the MCS at any visit (Supplementary Table S6). At 12 monthst, 6/10 (60%) adolescents and none of the adults rated their general health at least somewhat better than in the previous year (Supplementary Table S7).

The PedsQL total score did not significantly change over time. The subscale scores were lowest for school and physical functioning, and highest for social functioning. Significant improvements over time were seen for adolescents' school functioning only (*P* = 0.03) (Table 4 and Figure 3E). Except for the school and emotional subscale, all subscales showed significant differences between adults and adolescents at all visits (Supplementary Table S6).

**Figure 4:**
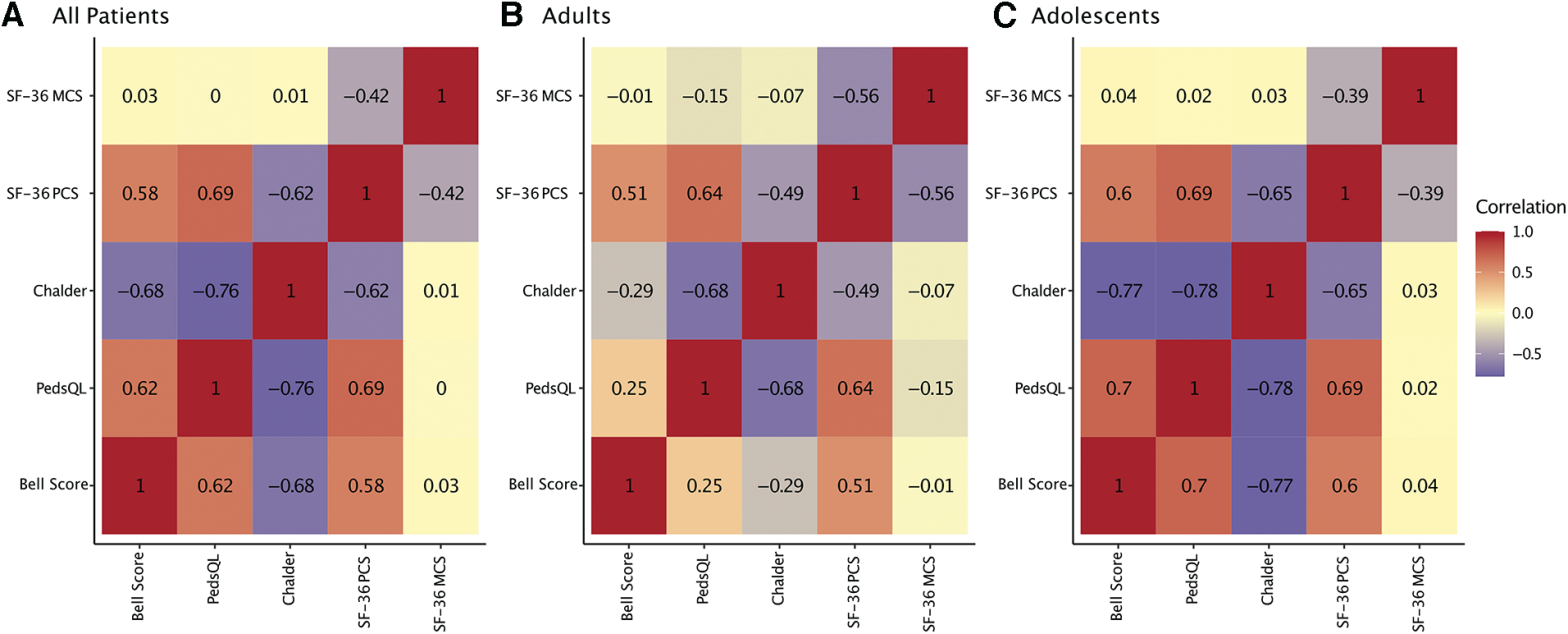
Correlation of patient-reported outcomes. Heatmap of repeated measures correlations between patient-reported outcomes (PROMs) for all patients (**A**), adults only (**B**), and adolescents only (**C**). Repeated measures correlations are a statistical tool to determine the overall within-patient correlation between a pair of variables.

For all patients best correlations among PROMs were found for CFQ and PedsQL (*r* = −0.76, *P* < 0.001), indicating that more severe fatigue was associated with lower HRQoL (Figure 4). The most prominent difference between adults and adolescents was that adolescents' but not adults' CFQ and Bell Score correlated significantly (adults: *r* = −0.29, *P* = 0.209; adolescents: *r* = −0.77, *P* < 0.001).

At 12 months, results from PROMs for patients in partial remission vs. no remission were median Bell Score 80 (range 40–100) vs. 40 (range 20–80), mean CFQ Score 12.4 (SD 6.7) vs. 24.4 (SD 6.2), PedsQL total score 76.1 (SD 16.8) vs. 46.6 (SD 15.8), SF-36 PCS 44.7 (SD 7.7) vs. 28.9 (SD 11.5), and SF-36 MCS 50.9 (SD 7.1) vs. 42.9 (SD 10.4). These results again indicate that patients with partial recovery might still suffer from impairment of daily life.

## 4 Discussion

This report contributes to rare follow-up data on young people with ME/CFS after EBV-IM. We present data on clinical phenotypes and HRQoL from a first German cohort of adolescents and young adults over time up to 12 months post ME/CFS diagnosis at our specialized tertiary pediatric center. So far, most data on pediatric and/or EBV-triggered ME/CFS originate from the US, the UK, and Australia, with no pediatric study from Germany (4, 6–8, 14–16, 26, 52–58). While some prospective pediatric studies examined ME/CFS with PEM after confirmed EBV-IM (17–19), to our knowledge, none compared adolescents and young adults with regard to symptom load and HRQoL over time.

### 4.1 Baseline demographics and ME/CFS diagnosis

Our youngest patient was 14 years-old, which was in line with the published ME/CFS age peak at 15–40 years (12, 46, 47). The observed female predominance (80%) is a widely recognized in post-pubertal ME/CFS patients (6, 20, 47). At the initial visit, all adults but only 8/12 (66%) met the CCC, supporting the use of more sensitive criteria for pediatric patients (52, 74, 75). Some pediatric follow-up studies employed the polythetic Fukuda criteria with the addition of mandatory PEM, while others used the broader Oxford criteria, potentially including individuals without ME/CFS (4, 7, 8, 14–16, 26, 53–58). To evaluate the CCC together with the CDW-R, the PCD-J, and the IOM criteria (47), we recently developed the Munich Berlin symptom questionnaire (MBSQ) (76).

The median diagnostic delay of more than one year was in line with most reports from other countries, indicating long and difficult patient journeys at any age (3, 52, 62, 63). We did not find any association of gender, age, or disease severity with time to diagnosis according to previous studies (62, 77). Published reasons for the diagnostic delay include insufficient knowledge by families and primary care providers, the requirement for comprehensive differential diagnosis, as well as negative attitudes and beliefs by primary care physicians and psychologists (52, 77, 78). A lack of ME/CFS specialists most likely exacerbates this issue. The young adults' longer disease duration prior to diagnosis possibly reflects challenges during transition from pediatric to adult health care services (79).

### 4.2 Postural tachycardia syndrome and other comorbidities

Comorbidities included PoTS (83%), allergies (48%), and psychiatric diagnoses (12%). The low prevalence of the latter alines with other reports on pediatric ME/CFS (6, 80, 81). PoTS has been reported in pediatric and adult ME/CFS cohorts with varying prevalence (6, 52). The large span of 5.7%–70% PoTS cases among adult ME/CFS patients (47) might in part be due to different PoTS tests and case definitions (82). Since PoTS is a frequent post-infectious phenomenon in adolescents (83) the high prevalence in our cohort was not unexpected. Since PoTS can significantly impair daily activities timely non-pharmaceutical and, if needed, pharmaceutical treatment is mandatory. In general, comorbidities are more prevalent in adult ME/CFS patients (79%–80%) (22, 81).

### 4.3 Lack of medical care

Only one of our patients had previously received a certificate of disability and none was supported by adequate medical devices or home care, reflecting poor medical care and barriers to specialized support (64–66). The large number of medical consultations prior to diagnosis, large proportion of our patients taking various dietary supplements and/or receiving complementary medical treatment, reflects the known lack of adequate, standard medical care and sets families at risk of financial challenges (7, 84).

All pupils in our study reported frequent school absences, and, remarkably, only a minority had received any educational assistance such as home or digital schooling. These findings align with earlier studies showing prolonged school absences and severely reduced social participation and education of young ME/CFS patients (3–8). This is particularly concerning, since pediatric patients with ME/CFS reported that remaining engaged in an education system that flexibly accommodated their illness and aspirations was crucial for their long-term functioning (7, 85, 86).

### 4.4 Laboratory findings

No established biomarker exist for ME/CFS, and standard laboratory tests typically yield unremarkable results (6, 52). Our patients mostly exhibited minor deviations, such as elevated ANA titers (56%), surpassing expectations for this age group (7). Elevated IgE levels were present in about a third, though previous studies found no clear associations with ME/CFS (87). Vitamin D deficiency was prevalent, yet it didn't seem directly linked to fatigue levels in another ME/CFS cohorts (88).

As expected, all patients showed anti-EBV VCA IgG as an indicator of previous EBV infection. Notably, undetectable EBNA-1 IgG and detection of anti-EBV VCA IgM, anti-EBV EA IgG, EBV DNA in throat washes weren't more common in our cohort than in the general population (89–92). Detectable EBV DNA in blood cells was more frequent than in a U.S. cohort without EBV-associated disorder (93). We found no significant correlation between EBV-specific results and disease severity or physical functioning, corresponding with earlier research that didn't establish a distinct pattern of EBV-specific virological results in ME/CFS patients (39, 41). However, our comprehensive EBV-specific immunological analyses suggest that EBV antigen mimicry might contribute to pathogenic autoimmunity (34, 43, 45, 94–98). While HHV, including EBV, are being discussed as potential causes or perpetuating factors of ME/CFS, no definite causal link has been established (39, 40, 41).

### 4.5 Partial recovery

The majority of our adolescent patients partially recovered after 12 months, while all adults still met the CCC. The different health trajectories were also evident in the self-perceived health transition item of the SF-36 at 12 months, with 40% and 20% of adolescents rating their general health as much better or somewhat better, and 45% and 22% of adults much worse or somewhat worse than in the previous year, respectively. Over the whole study period symptom load (see below) and school functioning (PedsQL) significantly improved in adolescents but remained stable or worsened in adults.

These findings are in line with compelling evidence indicating a better ME/CFS prognosis of children and adolescence compared to adults, with pediatric studies reporting recovery of up to 83% (4, 6–8, 14–16, 26, 49–58). Dramatic improvement was reported to be more likely within the first four years (6). Accordingly, partial remission in our cohort was associated with illness duration of less than 3 years. A systematic review indicated that prognosis in adults is fairly poor, with only a minority of adult patients experiencing full recovery (60).

Only two pediatric patients were largely symptom-free (except OI) at their last visit. Additionally, we noticed fluctuations of disease load over time, with some patients not meeting the diagnostic criteria at 6 but again at 12 months. Remissions and relapses are frequent in pediatric ME/CFS and can follow overexertion or additional infectious illnesses (6). Our findings support the recommendation that patients should be monitored closely and adviced even after partial recovery. However, it remains challenging to measure recovery from ME/CFS, especially in young people, since what they consider as “recovery” can largely differ (7) and effective pacing might mask ongoing disease (61).

### 4.6 Risk factors

Candidate risk factors affecting the prognosis of ME/CFS include age, female gender, fatigue severity at disease onset, PEM severity, severity of ME/CFS symptoms, comorbidities, illness duration, life stressors, and lower socioeconomic status (6, 14, 15, 78, 99, 100), although findings remained inconclusive. Our findings suggest younger age, shorter disease duration, a better Bell Score, and milder fatigue (CFQ) at initial presentation could potentially indicate a more favorable disease course in adolescents compared to adults. The small patient sample size prohibits definite conclusions. The interpretation of published data on ME/CFS outcome is challenged by the fact that in many ME/CFS cohorts the initial trigger is less well characterized than in our cohort (7, 8, 14–16, 26, 54, 56).

### 4.7 Symptom load and health-related quality of life over time

Patients experienced a wide range of persisting symptoms with little change in severity or frequency over time, showing interindividual variability and intraindividual fluctuations throughout the year. Pediatric ME/CFS symptoms typically fluctuate more than symptoms in adults (6). Adolescents reported fewer symptoms at 6 and 12 months while adults' symptom count remained steady. Adults consistently reported more symptoms and nearly double the frequency of adolescents. Quantifying frequency and severity of symptoms was recommended to increase the specificity of ME/CFS diagnosis (101), since mild symptoms are common in the general population. Our novel MBSQ can be use to quantify the severity and frequency of ME/CFS symptoms in a 5-point Likert scale (76).

Previous studies revealed that ME/CFS profoundly affects social life, education, and HRQoL of children and young adults, showing poorer HRQoL compared to peers with various other chronic diseases (7, 9–11). Notably, our adolescent cohort's PedsQL results closely resembled those from other countries, depicting similar HRQoL distributions (9, 10, 102, 103), with worse HRQoL in physical and school function and better results in social and emotional functioning.

Over time, adolescents showed moderate improvements in total, physical, and psychosocial score, particularly in the school domain, although social and emotional aspects remained stable. These improvements exceeded suggested clinically meaningful differences in pediatric cohorts (104). Intensive school counseling might have contributed to better school situations and HRQoL changes. We found little evidence of improved HRQoL in young adults, except for some gains in emotional and social subdomains, likely due to specialized care. Compared to adolescents, young adults in our cohort reported significantly lower HRQoL, which aligns with general findings on adult ME/CFS patients consistently demonstrating very low HRQoL (105, 106). The transition from pediatrics to adult patient medicine can be particularly challenging for young people with ME/CFS, with uncertainties regarding health care, education, financials, and contact to peers. Unrevealing age-specific risk factors will be crucial for developing effective preventive strategies.

Few studies have investigated HRQoL in adolescents with ME/CFS. Factors contributing to low HRQoL were identified as high frequency of PEM, cognitive symptoms, regular school absence, delayed school progression, and attending physical therapy or rehabilitation. School support and attendance, along with leisure activities, correlated with better HRQoL (9, 10). Contradictory findings exist about the impact of depressive symptoms (9, 10, 103, 106). ME/CFS criteria requiring PEM might select patients with worse HRQoL compared to polythetic criteria (10), and this might be especially true for the complex CCC used to diagnose ME/CFS in our adult patients.

### 4.8 Strengths and limitations

A strength of our study lies in providing long-term data on ME/CFS after serologically confirmed EBV-IM, supporting earlier reports on recovery (6, 7, 17). Confirming an infectious trigger of ME/CFS years later is challenging due to unreliable self-reports and to difficulties obtaining prior medical records. A second strength is the combined analyses of data from adolescents and young adults. The latter population often gets lost from pediatric as well as non-pediatric studies (107). Third and importantly, we provide data on ME/CFS cases that were diagnosed by clinical criteria requiring PEM as recommended by the European Network on ME/CFS research (EUROMENE) (46) and the Centers of Disease Control and Prevention (CDC) (79). Overall, our study adds to the current understanding of ME/CFS in young people and highlights the importance of an early diagnosis as well es of a thorough longitudinal evaluation of patients with ME/CFS following EBV-IM.

The study has limitations to be considered when interpreting the results. First, the low sample size and a potential selection bias limit the generalizability of results and may affect the statistical power. Second, although the drop-out rate of 20% at 12 months was deemed acceptable, it might contribute another bias. Third, the investigation of preexisting risk factors was limited, since patients were seen late after ME/CFS onset with potential recall bias. In addition, a longer follow-up period would be beneficial. Finally, the lack of a matched control group challenges the interpretation. Future studies with larger sample sizes, longer follow-up periods, and appropriate control groups are necessary to further validate and extend our findings.

### 4.9 Conclusions

In conclusion, ME/CFS after EBV-IM is a debilitating disease that results in severe functional impairment and poor HRQoL of both adolescents and young adults, with evidence of partial recovery in adolescents over time. Access to appropriate healthcare is a fundamental barrier for young people with ME/CFS in Germany as well as abroad. ME/CFS patients showed fluctuating symptoms, with adults reporting more symptoms, greater physical impairment, and worse HRQoL than adolescents. Laboratory findings did not provide any evidence for EBV replication perpetuating the disease. Further research is needed to clarify the responsible pathomechanisms, identify reliable biomarkers and risk-factors, and to develop effective strategies for ME/CFS treatments and prevention in young people.

## Data Availability

The raw data supporting the conclusions of this article will be made available by the others upon reasonable request.
